# Diagnostic Accuracy of Clinical Measures Considering Segmental Tissue Composition and Volume Changes of Breast Cancer-Related Lymphedema

**DOI:** 10.1089/lrb.2017.0047

**Published:** 2018-08-01

**Authors:** Eun Joo Yang, Seoung Yeon Kim, Woo Hyung Lee, Jae-Young Lim, Jaebong Lee

**Affiliations:** ^1^ Department of Rehabilitation Medicine, Seoul National University College of Medicine, Seoul National University Bundang Hospital, Seongnam-si, Korea.; ^2^ Department of Rehabilitation Medicine, Seoul National University Hospital, Jongno-gu, Korea.; ^3^ Medical Research Collaborating Center, Seoul National University Bundang Hospital, Seongnam-si, Korea.

**Keywords:** lymphedema, breast neoplasms, quality of life, arm

## Abstract

***Background:*** If we use only volumetry for measuring lymphedema, we could underdiagnose lymphedema with characteristics of biomechanical changes without definite volume change, especially in the medial forearm.

***Methods and Results:*** In total, 158 breast cancer patients participated in this study. Arm volume was measured by water displacement volumetry, and segmental volumes were calculated from circumferences by using the truncated cone method. Subcutaneous ultrasound echogenicities were assessed on the medial side of the upper arm and forearm of both arms and graded by subcutaneous echogenicity grade (SEG) and revised SEG (rSEG). The standards for diagnosing secondary lymphedema were according to the volume change and clinical stage. Sensitivity, specificity, receiver-operating characteristic (ROC) curve, and area under the curve (AUC) were used. Analysis of ROC curves yielded AUCs of 0.875–0.933 (*p* < 0.001). Volume differences in each segment were significantly different among the grades by SEG. The highest AUC was found for volume difference (AUC = 0.919, 95% confidence interval [CI] = 0.860–0.978) in the upper arm near the elbow; however, in the medial forearm, the highest AUC was found for rSEG (AUC = 0.948, 95% CI = 0.923–0.965 in the proximal forearm; AUC = 0.940, 95% CI = 0.923–0.965 in the distal forearm).

***Conclusions:*** Our findings support the use of SEG by ultrasound in the assessment of lymphedema, especially in the medial region of the forearm. Subcutaneous ultrasound echogenicities may improve the accuracy of diagnosis of lymphedema in the forearm.

## Introduction

Breast cancer-related lymphedema (BCRL) is a chronic, debilitating condition with a variety of causes that restricts the flow of lymphatic fluid.^1,2^ During the initial stages of lymphedema, the edema may be soft with pitting, and the severity of the condition is reflected by limb volume change. If left untreated, a reaction to the tissue injury induces the accumulation of inflammatory cells, a hallmark of a pathophysiological skin event. Staging is subjective and does not reflect the distribution of edema; more objective measures are required to delineate disease status more precisely.^3^

Based on the evidence in literature studies, there does not appear to be a gold standard for the formal grading or measurement of the severity of lymphedema. Although rarely identified as such, the frequency of use of different measures of limb volume or circumference would suggest that these measures are considered de facto gold standards for diagnosing secondary lymphedema. The accepted gold standards for measuring limb volume change in the clinical environment are water displacement volumetry and circumferential limb measures. The most common criterion for lymphedema diagnosis is a volume difference of ≥200 mL. Although limb volume is important and is currently the most objective clinical measure, measurement of volume alone may overlook important changes in tissue texture and the presence of latent lymphedema.^4^ Fibrotic changes in lymphedematous tissue are key in terms of progress.^5,6^ The clinical severity of lymphedema is graded according to the staging system of the International Society of Lymphology (ISL).^7^ Such staging is based on findings from a physical examination of the most severely affected part of the limb and, within each stage, an inadequate but functional severity assessment exists that assesses severity based on limb volume increases and trophic skin changes with fibrosis from baseline. According to the ISL classification, reversible stage (subsides with elevation) and irreversible stage (tissue swelling rarely decreases) can be classified and the irreversible stage is used as the gold standard for lymphedema in the United States and globally.^7^

Subcutaneous ultrasound echogenicity is an objective measure of the severity of lymphedema in the extremities.^3^ In the advanced stages of lymphedema, both volume and fibrotic changes clearly progress. Killaars et al.^8^ found a significant correlation between the difference in volume and the difference in elasticity in BCRL. However, the association of volume and fibrotic change may differ depending on the region of the lymphedema.

Recent research has suggested that specific regions of the arm may be affected first as lymphedema develops.^9^ Such regions would be exposed to such physiological changes for a longer period and may, therefore, exhibit greater changes in soft tissue composition compared with the surrounding regions. Modi et al.^10^ investigated the regional distribution of swelling in relation to local lymph drainage rates in the forearm; segmental variations were often evident.^11^ Czerniec et al.^12^ examined the segmental distribution of adipose tissue and showed that the soft tissue composition changes associated with BCRL may occur in the presence of pitting and predominantly affected the proximal forearm. This localization of adipose tissue may be because this region is affected early during the development of lymphedema.^13,14^ A comparison of echogenicity and volume data according to body part is required to assess the lymphedema more precisely. If we use only volumetry for measuring lymphedema, we could underdiagnose lymphedema with characteristics of biomechanical changes without definite volume change, especially in the medial forearm.

In this study, we explored the relationships between arm volume, bioimpedance spectroscopy (BIS) measurement of extracellular fluid (ECF) levels, and ultrasound data on skin and subcutaneous tissue of patients with secondary lymphedema in the upper extremities. The purpose of this study was to compare the diagnostic accuracy of volume calculated from circumference, BIS, echogenicity grade by ultrasonography, and clinical staging by using sensitivity, specificity, receiver-operating characteristic (ROC) curve, and area under the curve (AUC).

## Materials and Methods

### Ethical approval

The Human Research Ethics Committee at each institution where the study was conducted gave ethical approval. The study protocol was approved by the institutional review board of Seoul National University Bundang Hospital (IRB No. B-1110-138-008). The need for written informed consent from the participants was waived.

### Participants

Women at least 18 years of age, at least 6 months after treatment for unilateral breast cancer, and with or without lymphedema were recruited, retrospectively. Exclusion criteria were as follows: bilateral breast cancer, current upper-extremity infection, lymphangitis, or contraindications to BIS testing, such as a pacemaker, other in-built stimulator, or pregnancy.

### Procedures

We used a cross-sectional study design. All participants attended a single evaluation session in the SNUBH lymphedema clinic conducted by one experienced lymphedema practitioner. Lymphedema stage was determined by assessing reduction of swelling with elevation, pitting with pressure, and the presence of fibrotic changes, by using the International Society of Lymphedema classification.^15^

#### Arm volume measurements

Arm volume measurements (water displacement) were performed by using a graduated plastic cylinder (Supplementary Fig. S1; Supplementary Data are available online at www.liebertpub.com/lrb). The point 10 cm proximal to the olecranon process (in a straight line) was marked. The arm was then inserted into the cylinder, and the cylinder was filled with water to this mark. The water level was recorded, as was the level after the arm had been carefully removed from the cylinder. The difference between these two volumes was defined as the arm volume. The process was repeated for the contralateral arm. The interlimb volume difference (ILD) was the volume of the ipsilateral arm minus the volume of the contralateral arm. The contralateral arm, thus, served as a control.

In addition, arm volumes of both affected and unaffected arms were calculated from limb circumferences. Circumference measurements were performed with a tape measure beginning at 14 cm above, 7 cm above, 7 cm below, 14 cm below, and at the level of the medial olecranon. The volume of each of the four arm segments was calculated from circumferences by using the formula for a truncated cone^16^ (Supplementary Fig. S1).

Arm segments were designated as segment A (7–14 cm above the medial olecranon), segment B (at the level of to 7 cm above the medial olecranon), segment C (at the level of to 7 cm below the medial olecranon), and segment D (7–14 cm below the medial olecranon). ILDs for each segment were defined as follows and calculated: ILD-A (volume of the ipsilateral arm of segment A minus the volume of the contralateral arm of segment A), ILD-B (volume of the ipsilateral arm of segment B minus the volume of the contralateral arm of segment B), ILD-C (volume of the ipsilateral arm of segment C minus the volume of the contralateral arm of segment C), and ILD-D (volume of the ipsilateral arm of segment D minus the volume of the contralateral arm of segment D). The volumes of the four arm segments were summed to establish the total limb volume from 14 cm below to 14 cm above the medial olecranon (Supplementary Fig. S1). Volume-based cut-offs for each segment and the whole limb were determined based on normative data, taking arm dominance into consideration.^17^

#### ECF measurements

##### Bioelectrical impedance analysis (BIA)

The participant rested in a supine position on an examination couch with her arms slightly abducted for measurement of the whole arm (wrist to axilla) ECF by using a bioelectrical impedance analyzer (BIA). The impedances of the affected and unaffected arms were determined by using the Inbody S10 Biospace device (Model JMW140; Biospace Co. Ltd., South Korea). The electrodes were attached to both ankles for legs and thumbs and middle fingers for arms. The BIA estimates extracellular water measured within each whole arm by using conductivity differences between various tissues attributed to differences in biological characteristics. In total, 30 impedance measurements were obtained at six different frequencies (1, 5, 50, 250, 500, and 1000 kHz) at the following five locations: the right and left arms, the trunk, and the right and left legs. From these data, the inter-limb impedance ratio, corresponding to the ratio of ECF, was calculated on each side for the whole arm.^18^ The use of inter-limb ratios allowed the unaffected arm to act as an internal control, accounting for individual variations in the body composition of the women.

##### Ultrasonographic examination

The skin and subcutaneous tissue were scanned by using an ultrasound system fitted with an 11-MHz transducer. The scan points were as follows: 14 cm above (A), 7 cm above (B), 7 cm below (C), and 14 cm below (D) the medial olecranon of the medial part of the arm in the ventral position. The probe was placed transversely on each arm. Subcutaneous echogenicity was evaluated at each scan point. Full-thickness subcutaneous hyperechogenicity of any degree was termed “increased echogenicity.” The subcutaneous echogenicity grade (SEG) was defined as follows (Supplementary Fig. S2):

- Grade 0: no increase in subcutaneous echogenicity relative to the contralateral side. The subcutaneous fat layer was black.
- Grade 1: a diffuse increase in echogenicity relative to the contralateral side, but horizontal or obliquely oriented echogenic lines caused by connective tissue bundles can be seen.
- Grade 2: a diffuse increase in echogenicity. Echogenic lines are not identifiable.

The revised SEG (rSEG) was defined as follows:

- Grade 0: no increase in subcutaneous echogenicity relative to the contralateral side. The subcutaneous fat layer was black.
- Grade 1: a diffuse increase in echogenicity relative to the contralateral side, but horizontal or obliquely oriented echogenic lines caused by connective tissue bundles can be clearly seen.
- Grade 2: a diffuse increase in echogenicity relative to the contralateral side, skin, and subcutaneous layer can be divided by echogenicity, but echogenic lines cannot be seen.
- Grade 3: a diffuse increase in echogenicity. Echogenic lines are not identifiable.

Because echogenicity evaluation is subjective and is easily influenced by variations in B-mode gain, this was first adjusted to black by reference to normal subcutaneous fat from another part of the body.

#### Medical record review

Patient characteristics, including age, level of education, income, job, side of dominance/handedness, and treatment characteristics, including cancer stage, type of surgery, type of axillary procedure, and adjuvant therapy, including chemotherapy, radiotherapy, and hormone therapy, were reviewed by research nurses trained in rehabilitation surveys at Seoul National University Bundang Hospital.

### Statistical analysis

Statistical analyses were performed by using SPSS (ver. 18) and STATA (ver. 14.0; Stata Corp., College Station, TX) software. Means and SDs for interval data were obtained. Independent t tests were performed for normally distributed data. The χ^2^ test was used for nominal and categorical variables.

To dichotomize ILDs, the cut-off point for the diagnosis of lymphedema was ≥200 mL.^19^ The reference standards used for the calculation of sensitivity and specificity were differences of total volume and segmental volume. The cut-off values of ILD-A, ILD-B, ILD-C, and ILD-D were 43.6, 79.2, 95.5, and 123.0 mL, respectively.^7,17^

The clinical stage was divided into reversible stage (stages 0, 1) and irreversible stage (stages 2, 3, 4). The SEG was divided into reversible stage (Grades 0, 1) and irreversible stage (Grade 2). The rSEG was divided into reversible stage (Grades 0, 1) and irreversible stage (Grades 2, 3). In our study, the standards for diagnosing secondary lymphedema were according to the volume change and clinical stage (<2 vs. ≥2).^7,17^ Sensitivity represents the rate of true positives found by the index test, whereas specificity represents true negatives. Likelihood ratios were calculated. To provide insight into the performance of each assessment tool, we compared areas under the ROC curves. ROC curves are often used to evaluate the ability of a diagnostic test to discriminate between those with and those without the condition of interest. An advantage of ROC curves is the ability to display true positives and false positives at all cut-off levels for a diagnostic test, regardless of the decision threshold required for dichotomized sensitivity and specificity calculations. Sensitivity and one minus specificity data over a range of outcomes were used to construct the ROC curves, and AUC was calculated with 95% confidence intervals (CIs) for dichotomous and continuous variables. Higher AUC values indicate greater accuracy. An AUC of 1.0 represents perfect sensitivity and specificity; an AUC of 0.5 indicates an essentially worthless test.

## Results

In total, 158 women who underwent surgery for breast cancer participated in this study. Descriptive characteristics of the study population are shown in Table 1. The patients were relatively young, with a median age of 52.0 years. Most had tumors at stage 1 or 2. The surgery performed was a mastectomy in almost two-thirds of all cases.

**Table 1.** Descriptive Characteristics of the 158 Study Patients

	*Patients*
Age at diagnosis (years)	52.0 ± 11.0 (30–86)
Education	
Elementary school	14 (8.9%)
Middle school	8 (5.1%)
High school	55 (34.8%)
University	81 (51.3%)
Economic status	
Low	41 (25.9%)
Middle	64 (40.5%)
High	28 (17.7%)
Working	65 (41.1%)
Weight (kg)	60.9 ± 17.8
BMI	32.1 ± 61.8
Fat (%)	29.2 ± 7.9
Muscle (kg)	39.9 ± 4.9
Cancer stage	
T0	4 (1.5%)
T1	93 (59.1%)
T2	44 (28.8%)
T3	10 (6.1%)
T4	7 (4.5%)
N0	40 (25.4%)
N1	64 (40.3%)
N2	28 (17.9%)
N3	26 (16.4%)
Breast surgery	
Mastectomy	95 (60.3%)
BCS	63 (39.7%)
Axillary surgery	
ALND	112 (70.9%)
SLNB	46 (29.1%)
Neoadjuvant chemotherapy	78 (49.3%)
Radiotherapy	130 (82.1%)
Chemotherapy	134 (85.0%)
Hormonal therapy	106 (67.0%)

Values are presented as mean ± standard deviation or *n* (%).

ALND, axillary lymph node dissection; BCS, breast conserving surgery; SLNB, sentinel lymph node biopsy.

**FIG. 1.**
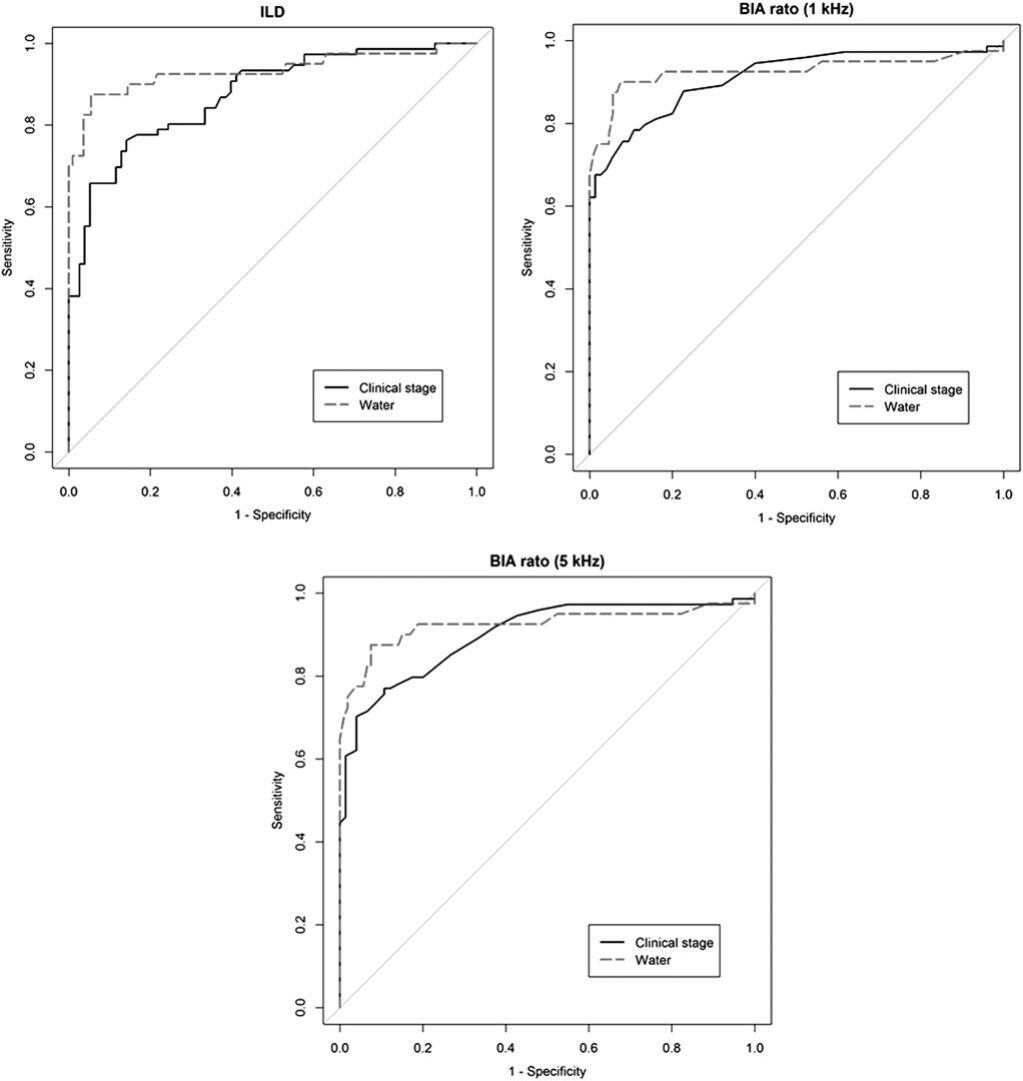
ROC curve of whole arm volume and lymphedema measurement tools. Arm volume measurements were measured by water displacement by using a graduated plastic cylinder, and the ILD was dichotomized with the cut-off point (≥200 mL) for the diagnosis of lymphedema (*water*). The clinical stage is divided into reversible stage (stages 0, 1) and irreversible stage (stages 2, 3, 4). BIA, bioelectrical impedance analyzer; ILD, interlimb volume difference; ROC, receiver-operating characteristic.

**FIG. 2.**
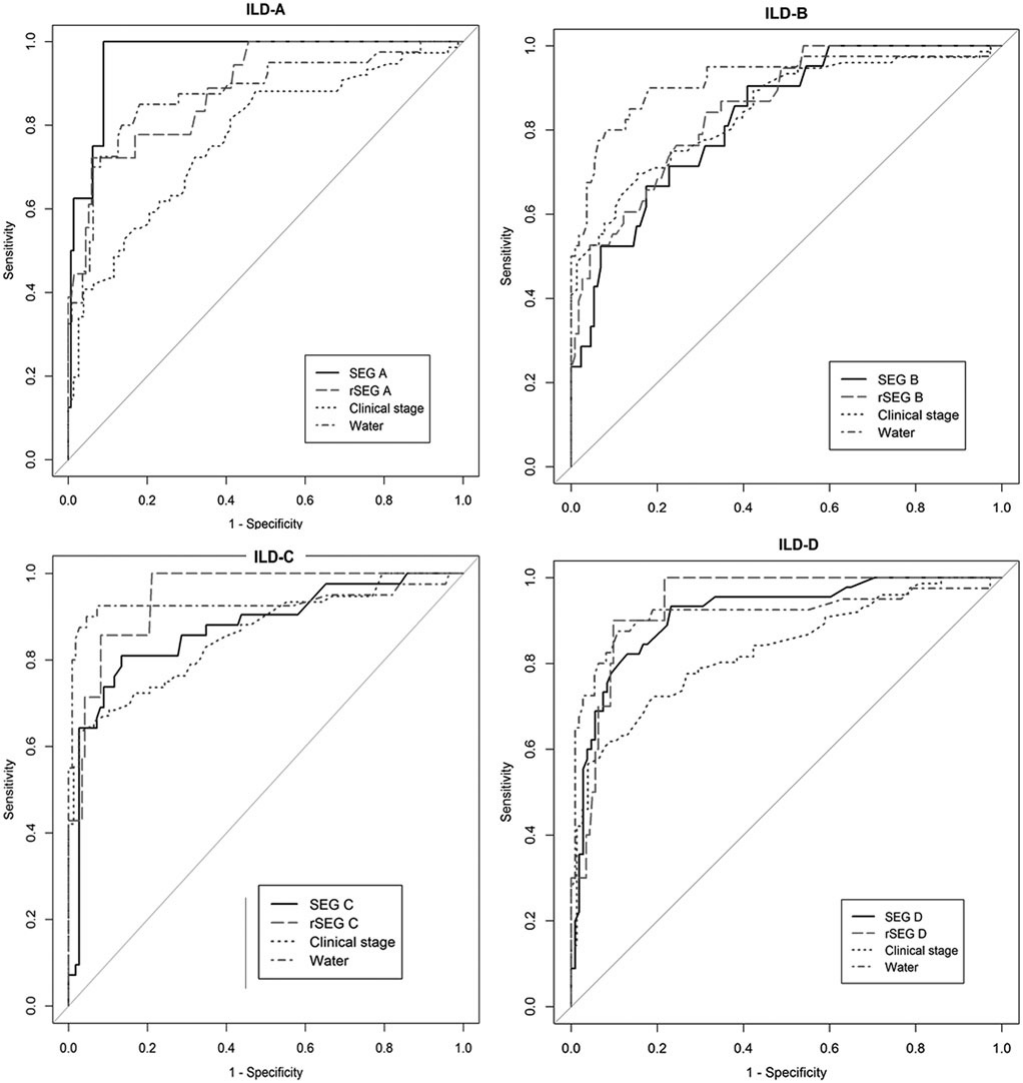
ROC curve of segmental volume and lymphedema measurement tools. Arm volume measurements were measured by water displacement by using a graduated plastic cylinder, and the ILD was dichotomized with the cut-off point (≥200 mL) for the diagnosis of lymphedema (*water*). The clinical stage is divided into reversible stage (stages 0, 1) and irreversible stage (stages 2, 3, 4). ILD-A: volume of the ipsilateral arm of segment A minus the volume of the contralateral arm of segment A (7–14 cm above the medial olecranon); ILD-B: volume of the ipsilateral arm of segment B minus the volume of the contralateral arm of segment B (level with to 7 cm above the medial olecranon); ILD-C: volume of the ipsilateral arm of segment C minus the volume of the contralateral arm of segment C (level with to 7 cm below the medial olecranon); and ILD-D: volume of the ipsilateral arm of segment D minus the volume of the contralateral arm of segment D (7–14 cm below the medial olecranon). The SEG was divided into reversible stage (Grades 0, 1) and irreversible stage (Grade 2). The rSEG was divided into reversible stage (Grades 0, 1) and irreversible stage (Grades 2, 3). SEG, subcutaneous echogenicity grade, rSEG, revised subcutaneous echogenicity grade.

Outcomes of physical measures used to assess lymphedema are presented in Table 2. Significant ILDs were found between groups according to the volume change and clinical stage. We also found significant differences in BIA ratio between the groups. For continuous data, the ROC curves are displayed in Figure 1, and analyses of ROC curves for the continuous outcomes yielded AUCs of 0.875–0.933 (*p* < 0.001) when the gold standard for lymphedema was defined by using clinical stage (<2 vs. ≥2)^7^ and the cut-off value for volume difference (<200 mL vs. ≥200 mL). When we used clinical stage as the gold standard, the highest AUC was found for a 1-kHz BIA ratio at 0.911 (95% CI = 0.862–0.960). If the gold standard was defined in terms of the volume difference, the highest AUC was found for ILD at 0.933 (95% CI = 0.874–0.992). Both BIA ratio and ILD had high AUC values (>0.9, Table 3). The ROC curve of segmental volume and lymphedema measurement tools are shown in Figure 2, and AUC data are presented in Table 3. In each segment, the gold standard for lymphedema was defined by using the cut-off value of each segmental volume difference.^17^ From each ROC curve for dichotomous outcomes, such as SEG and rSEG at each segment, clinical stage, and volume difference for the whole arm, we determined the diagnostic value with the highest AUC. In segment A, the highest AUC was for SEG at 0.966 (95% CI = 0.933–0.999). In segment B, the highest AUC was for volume difference (AUC = 0.919, 95% CI = 0.860–0.978). In segments C and D, the highest AUC was for rSEG (AUC = 0.948, 95% CI = 0.923–0.965 and AUC = 0.940, 95% CI = 0.923–0.965, respectively). The clinical stage of each segment (A, B, C, and D) had the lowest AUCs (AUC = 0.769, 95% CI = 0.695–0.844, AUC = 0.846, 95% CI = 0.785–0.907, AUC = 0.859, 95% CI = 0.791–0.922, AUC = 0.830, 95% CI = 0.721–0.897, respectively).

**Table 2.** Difference Between Groups in Outcomes of Lymphedema Assessment

	*Volume*		*Clinical stage*	
	*<200 mL (*n* = 111)*	*≥200 mL (*n* = 47)*	*<2*	*≥2*
BIA ratio (kHz)				
5	1.040 ± 0.926^a^	1.432 ± 0.338	1.005 ± 0.065^b^	1.282 ± 0.299
1	1.040 ± 0.912^a^	1.442 ± 0.354	1.004 ± 0.057^b^	1.289 ± 0.310
ILD (mL)	64.9 ± 97.7^a^	499.7 ± 307.3	34.6 ± 88.6^b^	312.7 ± 289.1
ILD-A	23.5 ± 36.2^a^	113.1 ± 76.2	17.7 ± 34.7^b^	72.7 ± 71.3
ILD-B	15.6 ± 25.4^a^	111.9 ± 75.4	8.6 ± 21.6^b^	71.2 ± 68.7
ILD-C	6.8 ± 20.6^a^	102.2 ± 66.2	0.9 ± 18.7^b^	61.4 ± 63.2
ILD-D	10.6 ± 26.5^a^	102.3 ± 66.2	4.3 ± 26.7^b^	63.1 ± 63.1

^a^ *p* < 0.05; Volume <200 mL versus Volume ≥200 mL.

^b^ *p* < 0.05; Clinical stage <2 versus Clinical stage ≥2.

BIA, bioelectrical impedance analyzer; ILD, interlimb volume difference; ILD-A, volume of the ipsilateral arm of segment A minus the volume of the contralateral arm of segment A (7–14 cm above the medial olecranon); ILD-B, volume of the ipsilateral arm of segment B minus the volume of the contralateral arm of segment B (at level −7 cm above the medial olecranon); ILD-C, volume of the ipsilateral arm of segment C minus the volume of the contralateral arm of segment C (at level −7 cm below the medial olecranon); and ILD-D, volume of the ipsilateral arm of segment D minus the volume of the contralateral arm of segment D (7–14 cm below the medial olecranon).

**Table 3.** Area Under the Receiver-Operating Characteristic Curve

*Index text*	*AUC (95% CI)*	p
Whole volume		
BIA resistance ratio (5 kHz)		
Clinical stage	0.900 (0.849–0.951)	<0.001
Water	0.924 (0.856–0.992)	<0.001
BIA resistance ratio (1 kHz)		
Clinical stage	0.911 (0.862–0.960)	<0.001
Water	0.927 (0.858–0.995)	<0.001
ILD		
Clinical stage	0.875 (0.821–0.929)	<0.001
Water	0.933 (0.874–0.992)	<0.001
Segment A		
SEG	0.966 (0.933–0.999)	<0.001
rSEG	0.891 (0.814–0.968)	<0.001
Clinical stage	0.769 (0.695–0.844)	<0.001
Water	0.878 (0.808–0.948)	<0.001
Segment B		
SEG	0.829 (0.741–0.917)	<0.001
rSEG	0.854 (0.789–0.919)	<0.001
Clinical stage	0.846 (0.785–0.907)	<0.001
Water	0.919 (0.860–0.978)	<0.001
Segment C		
SEG	0.872 (0.745–0.917)	<0.001
rSEG	0.948 (0.923–0.965)	<0.001
Clinical stage	0.859 (0.791–0.922)	<0.001
Water	0.933 (0.891–0.972)	<0.001
Segment D		
SEG	0.914 (0.874–0.972)	<0.001
rSEG	0.940 (0.920–0.962)	<0.001
Clinical stage	0.830 (0.721–0.897)	<0.001
Water	0.917 (0.865–0.988)	<0.001

Arm segments were designated as segment A (7–14 cm above the medial olecranon), segment B (at level −7 cm above the medial olecranon), segment C (at level −7 cm below the medial olecranon), and segment D (7–14 cm below the medial olecranon). Arm volume measurements were measured by water displacement by using a graduated plastic cylinder, and the ILD was dichotomized with the cut-off point (≥200 mL) for the diagnosis of lymphedema (*water*). The clinical stage is divided into reversible stage (stages 0, 1) and irreversible stage (stages 2, 3, 4).

AUC, area under the curve; CI, confidence interval; SEG, subcutaneous echogenicity; rSEG, revised subcutaneous echogenicity.

## Discussion

The purpose of this study was to evaluate the role of SEG for lymphedema assessment and to compare values obtained with this method with volume difference and clinical stage in each part of the arm. Using ultrasonography, a relatively fast and reproducible method, SEG parameters were shown to be comparable to those obtained by calculating volume differences. Importantly, this study demonstrated that revised grade categorization of subcutaneous echogenicity was capable of identifying lymphedema, especially in the forearm.

During the initial stages of lymphedema, the edema may be soft with pitting, and the severity of the condition is reflected in limb volume changes. A reaction to the tissue injury induces the accumulation of inflammatory cells, which is a hallmark of a pathophysiological skin event. Activation of lymphocytes and leukocytes and cytokine release, in turn, induce connective tissue synthesis by fibroblasts.^20^ Excessive extracellular matrix deposition, especially of collagen, constitutes the complex tissue response termed *fibrosis*. Changes in lymphedematous tissue are key in terms of the pathological progress of lymphedema.^6,20^

Any localized increase in the ECF level may initiate corresponding changes in soft tissue composition. In this study, we found that, in the forearm, soft tissue changes, indicating increased adiposity, assessed by ultrasonography, were more related to segmental volume change than the de facto gold standards, defined by the whole arm volume change and clinical stage. Increased fat echogenicity, with blurring of the interface between the subcutaneous fat and skin, has been previously reported in lymphedema patients.^21^

The prevalence of BCRL ranges from 5% to 42%.^22,23^ Differences in diagnostic criteria and the lack of a standardized assessment tool contribute to this variability. In clinical practice, lymphedema evaluation tends to focus on volume changes.^24^ In addition, bioimpedance instruments can be used to measure ECF levels.^25,26^ In this study, both BIA ratio and volume calculated from circumference demonstrated high AUC values, more than 0.9, if we use the criterion for lymphedema as a volume change of more than 200 mL. When dividing the whole arm into four segments, that criterion had the highest AUC value only in segment B (level with to 7 cm above the medial olecranon).

However, the accuracy of volume difference was lower than that of SEG in the forearm. Subcutaneous echogenicity is an objective measure of the severity of lymphedema of the extremities.^3^ Traditional methods of volume assessment yield no information on tissue composition.^27^ Pathophysiological changes in lymphedematous tissue are key in terms of the pathological progress.^5,6^ Ultrasound echogenicity has been reported to be useful in evaluating the clinical severity of lymphedema. Imaging techniques can assess tissue changes but are often not feasible; the instruments are expensive, and scanning costs are high. Suehiro et al.^3^ evaluated patients with secondary lower-extremity lymphedema and found that subcutaneous echogenicities, measured by using low-resolution ultrasound, were useful in evaluating lymphedema of the extremities. This is of practical utility to many physicians. Our findings of AUCs of 0.829–0.966 support the use of SEG in the assessment of BCRL. In this study, the subcutaneous echogenicities correlated well with both ISL stage and upper arm volume.

Stout et al.^14^ suggested that the very earliest changes during the development of lymphedema would likely be evident in superficial tissue lying close to muscles, primarily of the forearm and distal upper arm. Localized changes in soft tissue composition around the elbow may be in progress before volume is significantly increased. Even when the volume changes were relatively small, the affected forearm exhibited soft tissue changes.^28^ Tassenoy et al.^29^ noted postmortem adipose tissue changes even in the absence of visible swelling. Volume measurements alone are not sufficient to evaluate the progress and/or severity of forearm lymphedema. Tissue composition can change without an increase in volume. The clinical implication is that, even in cases of mild lymphedema (in the forearm), the presence of adipose tissue may hinder conservative efforts to reduce limb volume.

Previous studies noted variations in the segmental drainage patterns of the deeper subfascial compartments of the upper limb, most notably those of the muscle compartments and the hand. Anatomical differences in limb lymphatic drainage pathways have been identified radiographically^13^; segmental drainage pattern variances were evident in the deeper subfascial limb compartment, the forearm, and the hand. Segmental changes can be captured by optoelectronic infrared perometry,^10^ measurement of the circumferences of arm segments (yielding volume data), dual-energy X-ray absorptiometry-mediated assessment of tissue composition, and BIS of ECF.^12^ Unlike volumes, echogenicity thickening and disturbances can be measured directly by ultrasonography.^3^ An increase in the subcutis thickness of the ventral lower arm and associated changes in echogenicity are good indicators of lymphedema (sensitivity: 67%–100%). However, it is time-consuming to measure the thickness of the cutis and subcutis, and standardization is difficult because of individual variation. Suehiro et al.^3^ defined SEGs and found that these grades reflected the ISL stages of patients with secondary lower-extremity lymphedema. From a practical viewpoint, subcutaneous echogenicity grading is useful because it may be performed on any part of the extremity at any stage of the disease.^3^

Devoogdt et al.^30^ used ultrasonography to evaluate arms with and without lymphedema after axillary dissection to treat breast cancer. However, postoperative changes in echogenicity were not compared with extracellular arm fluid contents. In this study, we also used subcutaneous echogenicity grading to evaluate upper-extremity secondary lymphedema and found that such grading was both feasible and useful when evaluating BCRL.

Our study had several strengths. We performed multiple measurements. including volume, bioimpedance, and echogenicity of the upper limbs, providing a chance for a better understanding of the relationship among these different modalities with respect to BCRL in more than 150 patients. Previous studies^3,8,30^ used small sample sizes. The clinical course of lymphedema is highly variable, and to generalize relationships, a large sample size would be important. We standardized our mode of lymphedema evaluation to allow us to evaluate patients at each ISL stage comprehensively by using specific tools.

A limitation of our study is that the participants were not assessed at a fixed time point from the surgery. However, in this study, various assessments were performed at the same time. In addition, we did not measure segmental arm soft tissue compositions by using dual-energy X-ray absorptiometry or BIS.

## Conclusions

We described our lymphedema evaluation protocol by using stage-specific measurement tools and evaluated associations among these variables. Our findings support the use of SEG by ultrasound in the assessment of lymphedema, especially in the medial region of the forearm. Subcutaneous ultrasound echogenicities may improve the accuracy of diagnosis and may be used to monitor lymphedema progress and severity in the forearm.

Longitudinal changes and analyses of treatment effects should be assessed comprehensively at all stages of lymphedema. A comprehensive standardized tool is needed to this end.

## Supplementary Material

Supplemental data

Supplemental data
