# Supplementary material for: Diagnostic Accuracy of Clinical Measures Considering Segmental Tissue Composition and Volume Changes of Breast Cancer-Related Lymphedema
Source: Lymphat Res Biol. 2018 Aug 1;16(4):368–76. doi: 10.1089/lrb.2017.0047 (PMC6104249; doi:10.1089/lrb.2017.0047)
Supplement: Supplemental data [file Supp_Fig1.pdf]

Supplementary Data

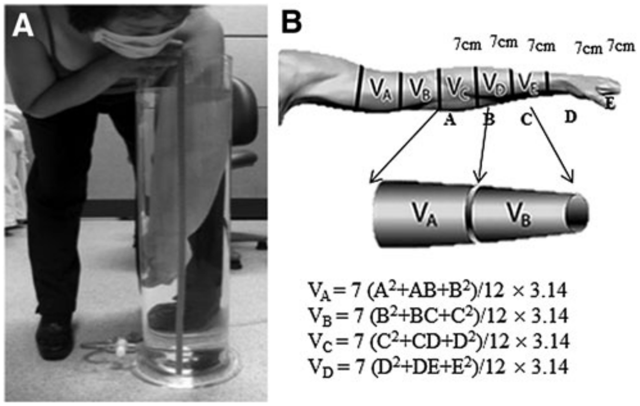

SUPPLEMENTARY FIG. S1. Arm volume measured by whole-arm water displacement volumetry **(A)** and by calculation from circumferences by using the truncated cone method **(B)**.
