# Supplementary material for: Diagnostic Accuracy of Clinical Measures Considering Segmental Tissue Composition and Volume Changes of Breast Cancer-Related Lymphedema
Source: Lymphat Res Biol. 2018 Aug 1;16(4):368–76. doi: 10.1089/lrb.2017.0047 (PMC6104249; doi:10.1089/lrb.2017.0047)
Supplement: Supplemental data [file Supp_Fig2.pdf]

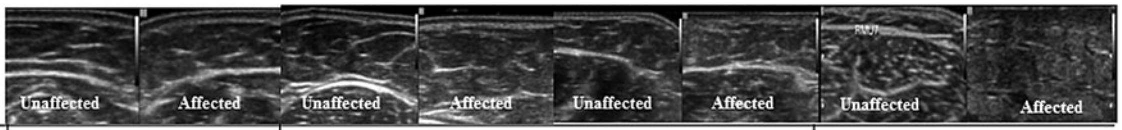


| **SEG** | **0** | **1** | | **2** |
|---|---|---|---|---|
| Echogenicity | Low | Increased | | Increased |
| Echogenic lines | Clear | Unclear but identifiable | | Unidentifiable |
| **rSEG** | **0** | **1** | **2** | **3** |
| Echogenicity | Low | Increased | Increased | Increased |
| Echogenic lines | Clear | Clear | Unclear but identifiable | Unidentifiable |

**SUPPLEMENTARY FIG. S2.** Definition of grade of SEG. Grade 0: No increase in echogenicity in the subcutaneous layer, Grade 1: Diffuse increase in echogenicity, horizontally or obliquely oriented echogenic lines caused by connective tissue bundles between skin and subcutis layer are unclear but identifiable, and Grade 2: Diffuse increase in echogenicity, echogenic lines are not identifiable. rSEG, revised subcutaneous echogenicity; SEG, subcutaneous echogenicity.
